# Dual role of microglia in glaucoma: Regulation of neuroinflammation and neuroregeneration

**DOI:** 10.4103/NRR.NRR-D-24-00876

**Published:** 2025-06-19

**Authors:** Panpan Li, Xin Shi, Verena Prokosch

**Affiliations:** 1 Department of Ophthalmology, Faculty of Medicine and University Hospital of Cologne, University of Cologne, Cologne, Germany; 2 Key Laboratory of Yunnan Province, Yunnan Eye Institute, Affiliated Hospital of Yunnan University, Yunnan University, Kunming, Yunnan Province, China; 3 Department of Orthopedics, The First People’s Hospital of Yunnan Province, Kunming, Yunnan Province, China; 4 The Affiliated Hospital of Kunming University of Science and Technology, Kunming, Yunnan Province, China

**Keywords:** glaucoma, inflammation, microglia, neurodegeneration, neuroregeneration, retinal ganglion cells

## Abstract

Globally, glaucoma stands as a primary cause of irreversible blindness, marked by intricate pathophysiological processes in which neuroinflammation plays a pivotal role. As the principal immune cells within the central nervous system, microglia play a dual function in the progression of glaucoma. Under standard physiological states, microglia safeguard the retina by offering neurotrophic support and removing cellular debris. In the pathological progression of glaucoma, microglia become activated and release significant levels of inflammatory factors, resulting in retinal ganglion cell injury, cell death, and impaired neuroregeneration. This review focuses on examining the dual functions of microglia in glaucoma, evaluating their influence on retinal neurodegeneration and repair, and suggesting that modulating microglial activity could serve as a promising therapeutic strategy. Understanding the mechanisms of microglial action in glaucoma is crucial for unveiling the complex pathophysiological processes of the disease and developing new therapeutic strategies.

## Introduction

Glaucoma is the leading cause of irreversible blindness for millions of people worldwide, with the number of glaucoma patients expected to reach 112 million worldwide by 2040 (Tham et al., 2014). This disease is characterized by the progressive loss of retinal ganglion cells (RGCs) and damage to the optic nerve, often associated with elevated intraocular pressure (IOP) (Casson et al., 2012). Although IOP-lowering treatments can mitigate disease progression, many individuals continue to experience vision deterioration, emphasizing that its pathophysiology extends beyond IOP elevation alone (Weinreb et al., 2014).

The pathogenesis of glaucoma is complex with multiple factors involved. Typically, elevated IOP is considered an independent significant risk factor (Sharfuddin Ahmed et al., 2023), which stems from impaired outflow of aqueous humor, leading to fluid accumulation and elevated IOP. Persistent high IOP imposes mechanical stress and induces ischemic damage, ultimately leading to RGC apoptosis (Weinreb et al., 2014). This specific cellular degeneration is driven by several interlinked mechanisms, including disrupted axonal transport, mitochondrial dysfunction, glutamate toxicity, and oxidative stress (Calkins, 2012). Moreover, the activation of glial cells and the subsequent release of inflammatory mediators exacerbate these effects, thereby accelerating the progression of the disease (Baudouin et al., 2021).

In the retina and optic nerve (ON) of glaucoma patients, it has been frequently documented that inflammatory cells and inflammatory mediators are upregulated (Tezel, 2013; Baudouin et al., 2021). Critical inflammatory mediators, such as tumor necrosis factor-alpha (TNF-α), interleukin-1 beta (IL-1β), and interleukin-6 (IL-6), play a key role in promoting neuronal damage and cell death. The inflammatory response associated with these factors not only exacerbates RGC damage and apoptosis but may also hasten the degeneration of the optic nerve.

Microglia, as the primary immune cells of the central nervous system (CNS), play a crucial role in immune monitoring, removing cellular debris, and modulating neuronal functions (Li and Barres, 2018). Under physiological conditions, microglia remain quiescent, whereas they can be activated following pathological conditions such as injury or disease, resulting in morphological and functional alterations accompanied by the release of various inflammatory factors (Paolicelli et al., 2022). While these factors can provide protection by addressing threats to the nervous system, they can also exacerbate neuronal injury and accelerate the development of neurodegenerative diseases (Prinz et al., 2019).

In glaucoma, microglia serve as the primary inflammatory cells, which are activated as the disease progresses that release a variety of inflammatory factors and mediators (García-Bermúdez et al., 2021). These inflammatory agents not only cause direct harm to RGCs but also worsen neurodegeneration by triggering additional inflammatory responses and activating related signaling pathways. Furthermore, researches have shown that excessive microglial activation is associated with impaired neuroregeneration in glaucoma (Ebneter et al., 2010; Guo et al., 2022).

Overall, the review seeks to explore the dual impact of microglia-driven inflammation in glaucoma, focusing on its role in both retinal neurodegeneration and regeneration. Through a detailed exploration of microglial mechanisms in glaucoma, this study aims to support the advancement of innovative therapeutic approaches for managing the disease.

## Search Strategy

The review covers studies published between 1994 and 2024, sourced from the PubMed database. The keywords employed in the search included “glaucoma,” “microglia,” “retina,” “RGC,” “neurodegenerative,” “neurodegeneration,” “neuroregeneration,” “neuroprotective,” “neuroinflammation,” “inflammatory,” and “inflammation,” independently and in combination. Articles were then screened for relevance based on abstract content. The review is intended to deliver a thorough overview of the dual regulatory roles and functions of microglia within the context of glaucoma.

## Microglia in the Retina

The retina is a complex multilayered organization comprising photoreceptors, ganglion cells, bipolar cells, and a variety of supporting cell types that are essential for supporting metabolism, removing waste products, and maintaining the balance of the local microenvironment (Wong-Riley, 2010). Within the retina, the resident glial cells—Müller cells, astrocytes, and microglia—each contribute critically to maintaining retinal integrity and overall health (Vecino et al., 2016). Microglia, the primary immune cells of the central nervous system, play a dual role of protection and destruction in maintaining homeostasis and responding to disease (Lloyd et al., 2019; Rodríguez-Gómez et al., 2020).

Retinal microglia typically perform active surveillance and immunological duties. Under the physiological state, microglia maintain retinal homeostasis by clearing debris and secreting neurotrophic factors. They constitute approximately comprise 5%–20% of the overall glial cells within the retina (Rathnasamy et al., 2019). Microglia initially exhibit an amoeboid morphology and appear at embryonic day 11.5, residing within the ganglion cell layer and neuroblast layer of the developing rodent retina, where they actively phagocytize apoptotic RGCs until postnatal day 5 (Santos et al., 2008). Subsequently, these microglia transition to a “ramified” phenotype with shrinking cytosol and prolonged branching, located in the plexiform layers under normal physiological conditions (Vecino et al., 2016). As highly motile surveillance cells, microglia migrate to injury sites and serve as the initial host defense system under pathological conditions (Wong-Riley, 2010). Their processes continually extend and retract to sense changes in the surrounding microenvironment (Nimmerjahn et al., 2005).

**Figure 1:**
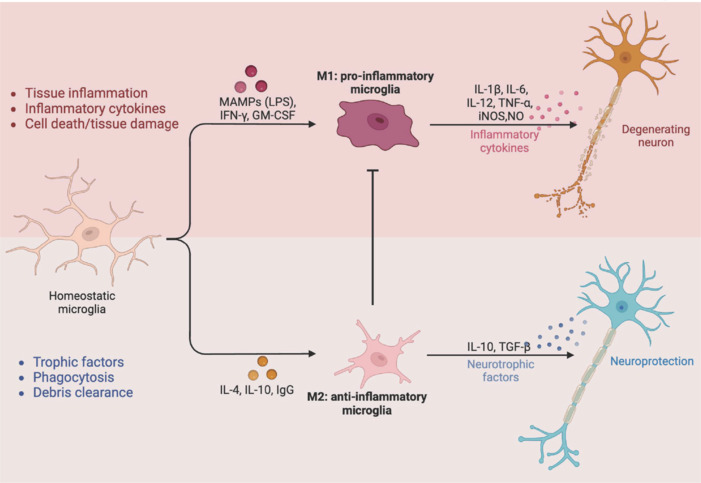
Two types of microglial activity. This image shows the transition of microglia from a ramified (resting) state under normal conditions to two amoeboid (activated) states, M1 and M2, illustrating their roles in surveillance and immune response. Created with BioRender.com. G-CSF: Granulocyte-colony stimulating factor; GM-CSF: granulocyte-macrophage colony-stimulating factor; IFN: interferon; IL: interleukin; iNOS: inducible nitric oxide synthase; LPS: lipopolysaccharide; MAMPs: microbe-associated molecular patterns; NO: nitric oxide; TGF-β: transforming growth factor beta; TNF: tumor necrosis factor.

Microglia may modify specific morphology and function depending on the dynamics of the endo-environment (Choi et al., 2021). Upon detecting tissue damage, ramified microglia transform into amoeboid microglia and colonize near lesions (Davalos et al., 2005). Activated microglia in their amoeboid state trigger a potent innate immune response, releasing inflammatory mediators classified as M1 (pro-inflammatory) and M2 (anti-inflammatory) cytokines, along with chemokines and neurotrophic factors, which further promote adaptive immune activation (**Figure 1**). The M1 state, characterized by neurotoxin activation and pro-inflammatory activity, produces cytokines such as TNF-α, IL-6, and IL-1β, which contribute to inflammatory responses (Kalkman and Feuerbach, 2016; Aguzzi and Zhu, 2017). As damage signals subside, microglia transition into an M2-like anti-inflammatory state, characterized by enhanced phagocytosis and the secretion of recovery-promoting molecules to facilitate tissue repair and clear cellular debris (Cherry et al., 2014; Park et al., 2016; Shemer et al., 2020; Yi et al., 2020). In an acute injury, manipulated stimulation of microglia mediates the neuroprotection restricting succesive histological damage, triggering regenerative processes to promote neural repair, thereby protecting retinal health. In conditions of persistent damage, including genetic mutations, prolonged oxidative stress, or hypoxia, microglial overactivation triggers the production of harmful inflammatory mediators associated with the M1 phenotype. This results in dysregulated inflammatory responses, thereby exacerbating neurodegenerative conditions (Rashid et al., 2019).

In conclusion, retinal microglia have dual functions: they play beneficial roles by providing neurotrophic support in homeostasis and harmful roles by exacerbating neuroinflammation in chronic pathological conditions. Aberrant innate immune activity within the eye is a key factor contributing to the development of retinal degenerative conditions. Retina-resident microglia revitalization is a co-feature of numerous retinal degenerative diseases (Langmann, 2007; Chen et al., 2018; Massengill et al., 2018; Yu et al., 2020).

## Neuroinflammation in Glaucoma

In glaucoma, long-term cumulative pressure increases can result in the loss of neuroprotective and re-modeling capacity of permanent neuroglia in the ganglion cell layer and/or the inner plexiform layer, release of apoptotic signals, degeneration of RGCs, and failure to regulate blood-retinal barrier integrity, resulting in neuroinflammatory degeneration (Tezel, 2011; Almasieh et al., 2012; Wang et al., 2016). These intricate processes are driven by interactions among glial cells and RGCs, mediated through both autocrine and paracrine signaling pathways (Danford et al., 2017).

Prolonged or fluctuating elevated IOP disrupts ocular blood flow, potentially causing vascular dysfunction, ischemic damage, and hypoxia, which in turn initiate acute or chronic inflammatory responses marked by the production of inflammatory mediators and infiltration of various immune cells (Flammer et al., 2002; Mozaffarieh et al., 2008; Vohra et al., 2013). Recruitment of microglia in the ganglion cell layer has been observed in hypoxic rat retinas (Kaur et al., 2015). When primary microglia cultures are exposed to hypoxic conditions, a significant rise in TNF-α and IL-1β levels is detected in the culture medium, enhancing apoptosis (Sivakumar et al., 2011). Endothelin-1 (ET-1), a powerful vasoconstrictor, plays a significant role in neuroglial activation, ischemic processes, and disruption of the retinal blood barrier, with its receptors contributing to the degeneration of RGCs and the optic nerve head (ONH) (Minton et al., 2012; Shi et al., 2024). ET-1 is principally derived from vascular endothelial cells, while partially originating from microglia/macrophages (Howell et al., 2011; Kowalczyk et al., 2015). Elevated plasma ET-1 levels in primary open-angle glaucoma patients are related to vasoconstriction, reduced ocular blood flow, and ischemia/hypoxia (Li et al., 2016). In DBA/2 J mice, ET-2 production in astrocytes and microglia/macrophages of the ONH occurs before retinal damage (Howell et al., 2014). Meanwhile, intravitreal administration of the ET-2 peptide could contribute to RGC axonal lesions (Howell et al., 2011). Deletion of NOX2 or inhibition by gp91ds-tat can mitigate injury to the inner blood-retinal barrier and neurovascular unit dysfunction, rescuing glaucoma-related RGC loss and ON axonal degeneration, associated with inhibition of ET-1/ERK1/2 pathway-induced microglial activation to pro-inflammatory M1 phenotype (Shi et al., 2024).

There is a close link between inflammation and extracellular matrix remodeling in the ONH. Matrix metalloproteinases (MMP) constitute a super-family of proteolytic enzymes that are architecturally homologous and cleave extracellular matrix ingredients comprising collagen, gelatin, and proteoglycans. Activated astrocytes in the ONH of glaucoma patients exhibit abnormal increases in MMP and tissue inhibitors of metalloproteinases (Agapova et al., 2001). Changes in MMP and tissue inhibitors of metalloproteinases are closely associated with inflammatory responses (Neumann et al., 2008). MMP-2 can cleave the transmembrane precursor of TNF-α from the membrane of glial cells, releasing soluble, bioactive TNF-α (De Groef et al., 2015).

Mitochondria are crucial for retinal homeostasis, and their dysfunction is an early driver of neuronal dysfunction (Williams et al., 2017). RGCs require optimal mitochondrial function to support cell survival (Osborne et al., 2008). Dysfunctional mitochondria release multiple damage-associated molecular patterns (DAMPs), such as adenosine triphosphate, reactive oxygen species (ROS), and mitochondrial DNA, activating the NLR family pyrin domain containing 3 (NLRP3) inflammasome and triggering proteolytic maturation of pro-inflammatory cytokine precursors, leading to nuclear factor ‘kappa-light-chain-enhancer’ of activated B-cells (NF-κB) pathway activation (Martinon, 2010). Retinas with intense mitochondrial activity are continuously exposed to ROS. ROS also serve as messenger molecules that drive the transcription of inflammation-related genes. Chronic oxidative stress is a major inducer of para-inflammation in aging retinas and a primary cause of para-inflammation in glaucoma (Xu et al., 2009; Nita and Grzybowski, 2016). Oxidative stress stimulates NF-κB, which inactivation in astrocytes facilitates the survival of retinal neurons post-ischaemic injury (Dvoriantchikova et al., 2009). NF-κB plays a crucial role in regulating the expression of various inflammatory molecules, including IL-6, TNF-α, inducible nitric oxide synthase, intercellular adhesion molecule-1, MMP-9, as well as apoptosis-inhibiting molecules like B-cell lymphoma 2 (Bcl-2) (Kabe et al., 2005; Harari and Liao, 2010). The antioxidant nuclear factor erythroid 2-related factor 2 (Nrf2) pathway is inhibited in glaucoma (Ahmed et al., 2017; Wang et al., 2020). Amplification of Nrf2 can depress the pro-inflammatory NF-κB pathway and facilitate neuron survival in neurodegenerative diseases and acute neural injuries (Xiong et al., 2015).

Within the development of glaucoma, massive amoeboid morphogenesis activating microglia aggregate around blood vessels in the lamina cribrosa and choroidal capillaries, indicating innate immune activation (Yuan and Neufeld, 2001; van Horssen et al., 2012; Zeng and Shi, 2018). A previous study has revealed high levels of pro-inflammatory cytokines and chemokines in the atrial fluid of glaucoma patients, implying that neuroinflammation plays a key role in glaucomatous neurodegeneration (Chua et al., 2012). In both human and experimental models of glaucoma, microglia are key players in sustaining neuroinflammation associated with the disease (García-Bermúdez et al., 2021), which responds to retinal injury, microglia are essential for preserving neuronal networks and mediating inflammatory responses within the neuroinflammatory processes of glaucoma (Chong and Martin, 2015; Colonna and Butovsky, 2017). In conclusion, a major contributor to the secondary degeneration of RGCs is the dysregulation of innate immune responses and the resulting neuroinflammatory activity (Tezel, 2013).

## Role of Microglial Activation in Neurodegeneration

### Release of inflammatory cytokines

As the resident immune cells within the nervous system, microglial cells usually remain in a quiescent state under normal, healthy conditions. However, with neurodegenerative diseases, the cells are activated and initiate the secretion of pro-inflammatory cytokines, such as TNF-α and IL-1β, which induce local inflammatory and neurotoxic responses that amplify neuronal damage. In glaucoma, the activation of microglia initiates inflammatory responses around RGCs, leading to direct RGC damage and hastening their progression toward apoptosis (Ji et al., 2024).

In various neurodegenerative diseases, the release of pro-inflammatory factors by microglia is a significant contributor to ongoing neuronal damage. A study by Smith et al. (2012) working with animal models revealed that in Alzheimer’s and Parkinson’s diseases, the sustained release of TNF-α and IL-1β not only activates additional glial cells but also intensifies inflammatory responses, creating a persistently neuroinflammatory environment. This cycle is recognized as a key factor driving neurodegeneration, as it fosters an irreversible inflammatory state that accelerates disease progression (Smith et al., 2012). Boccuni and Fairless (2022) further emphasize that pro-inflammatory factors from microglia have neurotoxic effects on the retina and disrupt the integrity of the blood–brain barrier (BBB), which can allow potentially harmful external substances to penetrate into the central nervous system, aggravating the neurodegenerative process.

### Programmed cell death and excitotoxicity

Beyond triggering inflammatory responses, activated microglia release pro-apoptotic factors that induce excitotoxicity and exacerbate RGC damage. In glaucoma, microglial activation leads to the secretion of pro-apoptotic molecules like TNF-α, which provokes the caspase-mediated apoptosis pathway in RGCs, ultimately resulting in cell death. Additionally, experimental research by Vernazza et al. (2021) shows that heightened microglial activation significantly accelerates RGC apoptosis through the regulation of caspase proteins, a process also documented in models of other neurodegenerative diseases (Vernazza et al., 2021).

Additionally, microglial activation enhances glutamate release, inducing excitotoxic effects in both the retina and the brain. This excitotoxicity activates N-methyl-D-aspartate receptors, causing an increase in intracellular calcium levels, which in turn triggers oxidative stress and mitochondrial dysfunction, ultimately leading to RGC apoptosis. Research by Guo et al. (2006) found that excessive glutamate release results in prolonged N-methyl-D-aspartate receptor activation, causing intracellular calcium overload and initiating apoptotic pathways in RGCs. A study on Alzheimer’s and Parkinson’s diseases by Ramirez et al. (2017) similarly showed that glutamate-induced excitotoxicity harms neurons through sustained activation of calcium channels, resulting in intracellular calcium overload and, ultimately, cell apoptosis.

### Disruption of the blood–brain barrier and blood–retinal barrier

Microglial activation can also worsen neuroinflammation by compromising the BBB and BRB. In glaucoma, Kim et al. (2016) observed that persistently activated microglia release pro-inflammatory factors and oxidative stress molecules, such as ROS and nitric oxide (NO), which weaken the tight junctions of the BRB, allowing toxic molecules to penetrate the retina and increase RGC damage (Hong et al., 2016). Kaur et al. (2013a) demonstrated experimentally that disruption of the BBB and BRB enables more harmful molecules to infiltrate the brain and retina, initiating a cycle of inflammation and oxidative stress that further drives neurodegeneration.

A similar mechanism of BBB disruption has been observed in neurodegenerative diseases such as Alzheimer’s disease. Saccà et al. (2020) found that BBB impairment permits toxic external molecules to enter the brain, initiating oxidative stress responses in neurons and causing prolonged neuronal damage. This barrier breakdown results not only leads to pro-inflammatory influences but also allows further infiltration of inflammatory mediators, amplifying neuroinflammation and accelerating the progression of degenerative pathology.

### Integrated neuroprotective strategies

As the understanding of microglial involvement in glaucoma and other neurodegenerative diseases expands, targeted modulation of microglial activity—specifically their pro-inflammatory and excitotoxic pathways—has become a promising therapeutic strategy. Tezel (2021) proposed that shifting microglial phenotypes toward an M2 anti-inflammatory state can significantly decrease the release of inflammatory cytokines, offering neuroprotection to RGCs and other neurons. This approach presents a viable intervention strategy for managing glaucoma and other neurodegenerative conditions.

Liu et al. (2022) further proposed that inhibiting the RIP1/RIP3/MLKL pathway attenuates microglia-induced excitotoxic injury, providing substantial protection for RGCs. In their glaucoma model experiments, selective inhibition of excitotoxic pathways was shown to alleviate calcium overload and oxidative stress, thereby preserving RGC integrity. These findings offer experimental support for the development of future therapeutic strategies aimed at glaucoma and other neurodegenerative diseases.

Collectively, microglial activation plays a critical role in driving neurodegeneration in glaucoma and various neurodegenerative diseases. This process initiates localized neuroinflammation through the release of pro-inflammatory cytokines, induces excitotoxicity due to excessive glutamate release, and disrupts both the BBB and BRB, allowing toxic molecules to infiltrate and accelerate disease progression. Therefore, strategies that regulate microglial activity, manage excitotoxic pathways, and protect the integrity of the BBB and BRB offer the potential to not only slow neuronal degeneration but also serve as promising therapeutic interventions for glaucoma and other neurodegenerative conditions.

## Dual Role of Activated Microglia in Glaucoma

The dual roles of microglia in glaucoma, encompassing both pro-inflammatory and anti-inflammatory effects, are summarized in **Table 1**, highlighting their impact on RGC damage and potential therapeutic strategies.

**Table 1:** Dual roles of microglia in glaucoma

Interventions	Mechanisms	Primary functions	References
Interferon-β	Modulates JAK-STAT pathway to inhibit pro-inflammatory cytokines (e.g., TNF-α and IL-1β)	Reduces neurotoxicity and inflammation	Dasgupta et al., 2002; Kawanokuchi et al., 2004
Dimethyl fumarate	Activates Nrf2 antioxidant pathway, suppresses M1 microglial activity	Promotes M2 polarization, alleviates neuroinflammation	Schilling et al., 2006; Ghoreschi et al., 2011
Glatiramer acetate	Modifies immune activity of microglia, reduces inflammatory cytokine release	Lowers neurotoxicity	Kim et al., 2004; Foster et al., 2007
Fingolimod (FTY720)	Regulates microglial activity states	Reduces cytokine release, mitigates inflammation	Kim et al., 2004; Foster et al., 2007
Mesenchymal stem cells	Secretes anti-inflammatory factors, promotes M2 polarization	Enhances neurotrophic support and neuroprotection	Laroni et al., 2013
TLR4/NF-κB pathway inhibition	Activation via DAMPs and PAMPs promotes M1 polarization	Reduces pro-inflammatory cytokines, mitigates neuroinflammation	Saijo and Glass, 2011; Eldahshan et al., 2019; Kumar, 2019
NLRP3 inflammasome inhibition	Modulates IL-1β and IL-18 secretion, promotes M1-to-M2 transition	Reduces neuroinflammation, protects neurons	Sui et al., 2020; Zhao et al., 2020
JAK/STAT pathway inhibition	Inhibits JAK2/STAT3 activation	Reduces pro-inflammatory response, alleviates neuroinflammation	Jones et al., 2015; Iwahara et al., 2017
AMPK and MAPK pathway activation	Improves metabolic status, promotes M2 polarization	Reduces inflammation, promotes tissue repair	Wang et al., 2018; Zhou et al., 2019; Plastira et al., 2020
miRNA regulation	Modulates transcription factors (e.g., CEBPα, PU.1) to influence microglial phenotype	Regulates inflammatory response, protects retinal ganglion cells	Fazi et al., 2005; Arora et al., 2013; Guedes et al., 2013; Butovsky et al., 2015

AMPK: 5& AMP-activated protein kinase; CEBP: CCAAT/enhancer‐binding protein; DAMPs: damage-associated molecular patterns; IL: interleukin; JAK: Janus kinase; MAPK: mitogen-activated protein kinase; NF-κB: nuclear factor ‘kappa-light-chain-enhancer’ of activated B-cells; NLRP3: NLR family pyrin domain containing 3; Nrf2: nuclear factor erythroid 2-related factor 2; PAMPs: pathogen-associated molecular patterns; STAT: signal transducer and activator of transcription; TLR: Toll-like receptor; TNF: tumor necrosis factor.

### Neurotrophic support provided by microglia in glaucoma

Within glaucoma patients, activated microglia at the ONH are associated with changes in cell morphology, protein expression, and antigen presentation (Yuan and Neufeld, 2001). Revitalized microglia phagocytose degenerated or dead RGCs to sustain a non-toxic retinal environment (Silverman and Wong, 2018). Retina-resident microglia and invaginated macrophages polarise into a neu-protective M2-like phenotype with enhanced phagocytosis, creating a microenvironment conducive to growth for axon regeneration following optic nerve crush (Raposo and Schwartz, 2014). Under the M2 phenotype, microglia release cytokines with anti-inflammatory properties along with neurotrophic factors (Wang et al., 2014; Tang and Le, 2016), which are inherently neuroprotective, promoting neuron survival and repair after CNS injury (Lipsky and Marini, 2007; Zhou et al., 2009). Increased IL-6 expression is a key component of the microglial response in glaucomatous retinas (Sappington and Calkins, 2006; Sappington et al., 2006; Echevarria et al., 2017). IL-6-deficient mice do not exhibit IOP-mediated deterioration of optic nerve architecture or consequent loss of vision implying that IL-6 probably plays a unique role in the development of RGC axonal degeneration and sight loss (Echevarria et al., 2017). IL-6 secreted by microglia significantly reduces apoptosis in RGCs damaged by hypertension, indicating an advantageous role of this cytokine in counteracting pro-apoptotic signal cascade in retinas (Sappington et al., 2006). Another study has reported that while IL-6 deficiency contributes to an anti-inflammatory, retina-survival-friendly environment, it also evokes an excessive TNF-α response under conditions of high IOP (Echevarria et al., 2016). TNF-α released by activated microglia induces astrocytes to produce neuroprotective factors following relatively mild hypertensive glaucoma injury (Lee et al., 2014). TNF-α participates in complex interactions between pro-inflammatory, pro-apoptotic, and pro-survival pathways mediated by two different receptor types, TNFR1 and TNFR2 (Ortí-Casañ et al., 2019). Intraocular pre-injection of TNF-α attenuates RGC destruction elicited from optic nerve crush in mice, implying that TNF-α exerts a protective effect during the initial phase of the RGC mortality process (Mac Nair et al., 2014). This effect may be facilitated through TNFR2, which is expressed by microglia, leading to the production of anti-inflammatory cytokines such as granulocyte-colony stimulating factor and IL-10 upon activation (Veroni et al., 2010).

Ciliary neurotrophic factor (CNTF) is known to be expressed by microglia (Harada et al., 2002; Tanaka et al., 2013). Research has demonstrated that CNTF stimulates rat microglia to release glial cell line-derived neurotrophic factor (Krady et al., 2008), indicating that CNTF influences glial cells to support motor neuron survival (Mitsumoto et al., 1994; Sagot et al., 1995). Intraretinal injection of CNTF appears to recruit activated retinal microglia and infiltrating macrophages, which enhances the survival of RGCs and promotes axonal regeneration following optic nerve injury in adult rats (Cen et al., 2007). Reportedly, the signal transducer and activator of transcription 3 (STAT3) is stimulated by CNTF in RGCs via the Janus kinase (JAK)/STAT signaling cascade, which may directly support the neuroprotective effects of CNTF on RGCs (Cui et al., 1999; Peterson et al., 2000). Deleting either CNTF or leukemia inhibitory factor, deleting both, or deleting STAT3, eliminates the neuro-regenerative state induced by inflammatory stimuli such as lens injury or zymosan treatment in the eye (Müller et al., 2007; Leibinger et al., 2013).

The lens injury reactivates retinal microglia, astrocytes, and Müller cells, as well as engages peripheral myeloid cells recruited into the retina, which brings adult RGCs into an active growth state, thereby enhancing the survivability of RGCs and axonal recovery and regeneration following optic nerve crush in both mice and rats (Leon et al., 2000; Fischer et al., 2001; Lorber et al., 2005). The body of research collectively shows that the secretion of specific factors plays a role in enhancing growth responses following lens injury by stimulating neuroglia, like microglia (Cui et al., 2009). The regenerative effects of lens injury can be mimicked by the pro-inflammatory agent zymosan, a yeast cell wall preparation (Yin et al., 2003). Dectin-1, a pattern recognition receptor for zymosan, is detected in permanent microglia and immersed macrophages in the retina (Tsoni and Brown, 2008). Dectin-1 is a key mediator of axonal regeneration in adult rat RGCs after optic nerve crush induced by zymosan (Baldwin et al., 2015). Within the retina, intravitreal injection from curdlan, an insoluble β-glucan complexing with dectin-1, allowed the enrollment of microglia and infiltrating myeloid cells overexpressing dectin-1 into the retina, thereby eliciting vigorous axonal rejuvenation over 2 weeks following retinal injury (Li et al., 2011).

In one recent investigation, supplement activity in residual retinal microglia and invasive macropositive cells was shown to be critical for successful axonal regeneration after spinal cord injury. Knockdown of the complement components C1q, C3, and CR3 receptors resulted in total failure of the growth-stimulating action of almost all of several interventions known to promote axonal regeneration after optic nerve crush in adult mice (Peterson et al., 2021), including zymosan-induced intraocular inflammation (Leon et al., 2000; Yin et al., 2003, 2009), blocking the accumulation of mobile zinc in injured RGCs with the zinc chelator N,N,N′,N′-tetrakis(2-pyridinylmethyl)-1,2-ethanediamine (Li et al., 2017), and treating with the cancer-regulating protein deleting phosphatase and tensin homolog (PTEN) (Kurimoto et al., 2010; de Lima et al., 2012). In mice, deletion of PTEN in RGCs enhances the survival and regenerative potential of α-RGCs (Duan et al., 2015), while stromal cell-derived factor selectively induces axonal regeneration of non-α-RGCs. Combining stromal cell-derived factor 1, oxyntomodulin, and PTEN deletion synergistically enhances the axonal regeneration of both α and non-α-RGCs (Xie et al., 2022). Resident microglia are the main source of retinal complement proteins C1q and C3 (Stevens et al., 2007; Schafer et al., 2012). Once activated and expressing elevated levels of the CR3 receptor, retinal microglia actively remove myelin debris from lesion areas following optic nerve crush, creating a growth-permissive microenvironment for axonal regeneration (Peterson et al., 2021).

During stress responses, microglia also secrete neurotrophic factors such as brain-derived neurotrophic factor (BDNF) to repair damaged tissue (Quigley et al., 2000; Parkhurst et al., 2013). BDNF belongs to the neurotrophin family, capable of inducing neuronal differentiation of stem cells, exerting anti-inflammatory effects, and improving the microenvironment of the CNS (Ge et al., 2019; Wang et al., 2019). A single intravitreal injection of BDNF protects RGCs across the entire retina in axotomized mice (Galindo-Romero et al., 2013). Additionally, reactive microglia express the anti-inflation biochemical cytokine transforming growth factor beta, and the high expression of transforming growth factor beta indicates that microglia seek to downregulate the degressive response in retinopathy in glaucoma (Yuan and Neufeld, 2001). Depletion of microglia in aged (9–12 months old) DBA/2J (D2) mice using PLX5622 significantly increases moderate to severe optic nerve damage with age-associated elevated IOP (Diemler et al., 2024). Overall, decreased microglia exacerbate neurodegeneration in glaucoma in D2 mice, demonstrating an overall benefit of microglia in preventing the reduction of RGCs linked to elevated IOP (Diemler et al., 2024). Therefore, glial reactivity is not inherently harmful but can produce beneficial effects when controlled (Alqawlaq et al., 2019).

### Inflammatory responses induced by activated microglia in glaucoma promote retinal damage

**Figure 2:**
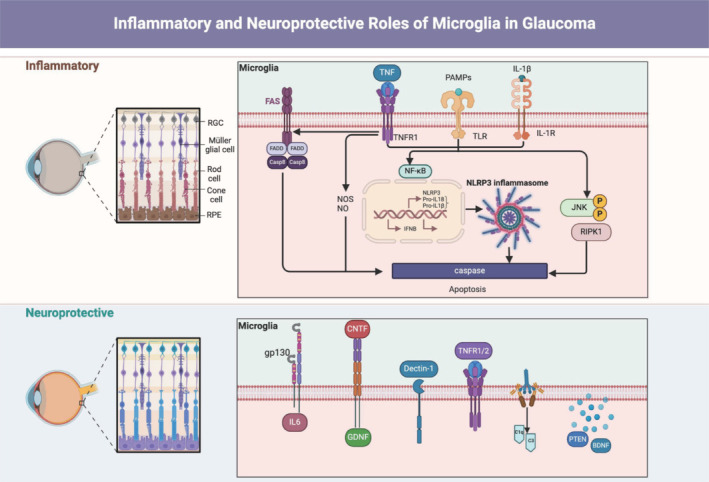
Inflammatory and neuroprotective roles of microglia. This image illustrates the dual roles of microglia in glaucoma, showing their involvement in both inflammatory responses that promote retinal damage and neuroprotective functions that support retinal health. Created with BioRender.com. BDNF: Brain-derived neurotrophic factor; CNTF: ciliary neurotrophic factor; GDNF: glial cell line-derived neurotrophic factor; IFN: interferon; IL: interleukin; JNK: c-Jun N-terminal kinase; NF-κB: nuclear factor ‘kappa-light-chain-enhancer’ of activated B-cells; NLRP3: NLR family pyrin domain containing 3; NO: nitric oxide; NOS: nitric oxide synthase; PAMPs: pathogen-associated molecular patterns; PTEN: phosphatase and tensin homolog; RGCs: retinal ganglion cells; RIPK1: receptor-interacting protein kinase 1; RPE: retinal pigment epithelium; TLR: Toll-like receptor; TNF: tumor necrosis factor.

Regardless of specific polarization states, microglial activation is a significant feature of neuroinflammation, prominent in nearly all neurodegenerative diseases (Ransohoff, 2016; Colonna and Butovsky, 2017). Inflammation is a pleocytotic cellular process in which immunocytes fight pollutants and mend damage through a variety of muscular and cytological changes. These changes include the secretion of pro-inflammatory cytokines, alongside mechanisms related to cell death through apoptosis and necrosis (Wallach et al., 2014). DAMPs produced by necrotic cells, such as IL-1α, TNF-α, and high-mobility group box 1 (Wallach et al., 2014), significantly enhance inflammatory responses (**Figure 2**). Inflammatory events are the most likely cause of progressive retinal degenerative disease (Whitcup et al., 2013), with activated microglia releasing cytokines and chemokines that lead to progressive apoptosis of photoreceptor cells (Arroba et al., 2018).

DBA/2J (D2) mice, which serve as a model for chronic inherited glaucoma with natural mutations in tyrosinase-related protein 1 and glycoprotein nmb, display activation and proliferation of microglia preceding the onset of noticeable RGC neurodegeneration, indicating microglial activation actively participates in the onset and progression of glaucomatous RGC neurodegeneration (Inman and Horner, 2007; Bosco et al., 2011). DAMPs released by RGCs or optic nerve head astrocytes can trigger inflammatory responses, confirmed to be a reaction to elevated IOP (Wei et al., 2019). Once microglia detect DAMPs, they transform into an M1-like pro-inflammatory phenotype, adopting highly motile amoeboid forms (Chen and Xu, 2015). Persistent microglial activation can lead to necroptosis, a form of programmed cell death mediated by the activation of receptor-interacting protein kinases 1 and 3 (RIPK1 and RIPK3) (Weinlich et al., 2017; Galluzzi et al., 2018). This necroptotic process subsequently releases pro-inflammatory DAMPs, fueling ongoing inflammation (Rodríguez-Gómez et al., 2020). Studies have shown that RIPK1/3-driven microglial necroptosis exacerbates neuronal injury (Welser et al., 2010; Ofengeim et al., 2017; He et al., 2021).

Chronic microglial activation leads to the release of neurotoxic and pro-inflammatory agents (Wang et al., 2016). In rodent models of glaucoma, including those subjected to hypoxic conditions or increased IOP, activated microglia release cytokines such as TNF-α and IL-1β, correlating with RGC apoptosis and highlighting microglial involvement in glaucomatous neurodegeneration (Sivakumar et al., 2011; Jassim and Inman, 2019). Evidence suggests that microglia contribute to RGC degeneration, partly through substantial TNF-α secretion following optic nerve crush in mouse models (Tezel et al., 2004). In hypertensive glaucoma rat models, elevated IOP rapidly elevates TNF-α levels from microglia around the ONH within days, initiating axonal degeneration and leading to a 38% RGC loss over subsequent weeks (Roh et al., 2012). TNF-α can impact RGC viability via a multitude of mechanisms, such as by activating NOS as well as NO expression and generation (Yuan and Neufeld, 2000), causing dysfunction of mitochondria (Kaur et al., 2013b), and evoking the expression of Fas ligand on membranes of retinal microglia and/or membranes of invasive macropinocytes (Krishnan et al., 2016). Additionally, TNF-α regulates tissue remodeling by promoting the syndrome and production of MMPs, among them MMP-9 (Shubayev et al., 2006), and stimulates the production of ET-1 in various intraocular cell lines (Desai et al., 2004).

TNF-α and one from its receptor, TNFR1, are overregulated in RGCs and retinal glial cells of glaucomatous retinas (Yuan and Neufeld, 2000). Soluble TNF-α binds to TNFR1 receptors on RGCs, inducing apoptosis through caspase activation (caspase 8), mitochondrial breakdown of membrane potential, and ROS generation (De Groef et al., 2015). These events are in keeping with the elevated levels of TNFR1 found in the inner layers of the retina in glaucoma patients (Yan et al., 2000; Yuan and Neufeld, 2001), and RGC death is significantly reduced by neutralizing TNF-α with antibodies (Tezel and Wax, 2000). In a mouse model of glaucoma, elevated IOP activates TNFR2 in microglia, with simultaneous reduction in oligodendrocytes in the further decline of RGCs in the optic nerves (Nakazawa et al., 2006). Inhibiting TNF-α activation with susceptible TNF-α receptor antagonists suppresses microglial responses and prevents axonal degradation and RGC loss in hypertensive glaucoma rat models (Roh et al., 2012). Blocking TNF-α minimizes the harmful effects of TNFR1 activation while preserving TNFR2-mediated signaling (Vargas et al., 2015).

TNF-α additionally increases the expression of the Fas ligand on microglial membranes and activates the Fas receptor on RGCs, resulting in apoptosis through caspase-8 activation (Baudouin et al., 2021). Recent reports suggest that TNF-α can replace LPS as an initiating signal, upregulating NF-κB to activate the NLRP3 inflammasome, leading to inflammasome-dependent IL-1β production in human primary macrophages (Magni et al., 2012; Jämsen et al., 2020). Pro-IL-1β and NLRP3 assemble into a distinctive heptameric structure, forming a large protein complex known as the NLRP3 inflammasome (Halle et al., 2008). This inflammasome activates caspase-1, leading to the production of pro-inflammatory cytokines IL-1β and IL-18, which can harm RGCs. Additionally, the c-Jun N-terminal kinase pathway is recognized as a primary mechanism in retrograde RGC injury, where damage begins in the axon and leads to cell apoptosis. Cytokines TNF-α and IL-1 released by M1 microglia activate c-Jun N-terminal kinase, further driving RGC apoptosis (Syc-Mazurek and Libby, 2019). Blocking TNF-α activity with the clinically approved drug etanercept has been shown to reduce inflammation in microglia, thereby decreasing optic nerve axonal degeneration and RGC loss (Roh et al., 2012). Overall, sustained inflammatory responses mediated by TNF-α and IL-1 may play a significant role in the retrograde injury pathway of RGCs (Syc-Mazurek and Libby, 2019; Ishikawa et al., 2023).

Toll-like receptor 4 (TLR4), located on the plasma membrane of microglia, is linked to normal tension glaucoma and primary open-angle glaucoma due to single nucleotide variations in the TLR4 gene (Takano et al., 2012). In experimental animal models of glaucoma, LPS injection activates microglia through TLR4 signaling and complement upregulation, exacerbating RGC damage (Astafurov et al., 2014). In an acute IOP-induced ischemia-reperfusion mouse model, IOP induces increased expression of TANK-binding kinase 1, promoting the ubiquitination and degradation of adenosine triphosphate binding cassette subfamily A member 1, reducing membrane localization of ANXA1 translocation and microglial activation, leading to RGC apoptosis (Li et al., 2018). Additionally, activated microglia release ROS and prostaglandin E2, leading to RGC apoptosis (Gao et al., 2013). Elevated inducible nitric oxide synthase and NO levels in the ONH of glaucoma patients (Neufeld et al., 1997). In experimental rat glaucoma models, microglial activation increases inducible nitric oxide synthase expression, NO production, and RGC damage (Hvozda Arana et al., 2020). Notably, the pharmacological inhibition of NO synthesis through the use of timolol significantly inhibits RGC degeneration in elevated IOP rats, suggesting that mechanisms mediated by NO result in neuronal cell death in glaucoma (Vidal et al., 2006). The connection between neuroinflammatory responses of microglia and RGC damage indicates that targeting neuroinflammatory pathways linked to microglial activation could offer neuroprotective benefits (Tan et al., 2020; Ishikawa et al., 2023).

### Therapeutic implications of microglial phenotypic modulation in glaucoma

In glaucoma, an imbalance between M1 and M2 microglial populations intensifies RGC damage, contributing to faster disease progression. **Table 2** outlines the specific roles of M1, M2, and mixed microglial phenotypes in neuroinflammation, demonstrating how modulating these phenotypes can impact both RGC survival and the management of inflammation in glaucoma.

**Table 2:** Roles and therapeutic potential of M1, M2, and mixed microglial phenotypes in neuroinflammation

Phenotypes	Characteristics	Primary functions	Mechanisms	Therapeutic potentials	References
M1 microglia	Pro-inflammatory	Release of inflammatory cytokines, exacerbating RGC damage	**Cytokine release:** Secretion of TNF-α, IL-1β, and IL-6 activates pro-inflammatory pathways such as NF-κB, causing neuronal damage and apoptosis **ROS production:** Generation of ROS induces oxidative stress, further damaging RGCs	M1 inhibitors (e.g., TNF-α inhibitors) could reduce neuroinflammation and protect RGCs	Tezel, 2013; Ransohoff, 2016; García-Bermúdez et al., 2021
		Induction of programmed cell death and excitotoxicity	**Excitotoxicity:** Release of glutamate leads to overexcitation of neurons, causing excitotoxicity and cell death **Apoptosis induction:** Fas/FasL pathway induces RGC apoptosis	Blocking M1 pathways or excitotoxic receptors (e.g., NMDA receptors) to mitigate neural damage	Calkins, 2012; Krishnan et al., 2016; Baudouin et al., 2021
M2 microglia	Anti-inflammatory	Release of anti-inflammatory cytokines, suppressing excessive inflammation and promoting tissue repair	**Anti-inflammatory cytokine secretion:** Release of IL-10 and TGF-β reduces pro-inflammatory responses and limits neuronal damage **Enhanced phagocytic function:** Clears retinal debris and inflammatory mediators, stabilizing retinal environment	M2 activators (e.g., IL-4, IL-13) could promote M2 polarization and reduce inflammation	Yuan and Neufeld, 2001; Silverman and Wong, 2018; Shemer et al., 2020; Yi et al., 2020
		Secretion of neurotrophic factors, supporting neuron survival and regeneration	**Neurotrophic support:** Release of CNTF and BDNF promotes RGC survival and axonal regeneration **JAK/STAT pathway activation:** CNTF stimulates STAT3 signaling, supporting neuroprotection in the retina	Promoting M2 polarization or direct administration of neurotrophic factors to stimulate nerve regeneration	Peterson et al., 2000; Harada et al., 2002; Müller et al., 2007; Wang et al., 2014
Mixed M1/M2 phenotype	Dynamic plasticity	Transition between pro-inflammatory and anti-inflammatory states to balance neural damage and repair	**Phenotypic plasticity:** Microglia shift between M1 and M2 states in response to environmental signals **Tissue damage cues:** Phenotype changes dynamically with tissue injury and repair signals	Modulating microenvironmental cues to control microglial polarization, achieving targeted therapeutic responses	Prinz et al., 2019; Paolicelli et al., 2022

BDNF: Brain-derived neurotrophic factor; CNTF: ciliary neurotrophic factor; FasL: Fas ligand; IL: interleukin; JAK: Janus kinase; NF-κB: nuclear factor ‘kappa-light-chain-enhancer’ of activated B-cells; NMDA: N-methyl-D-aspartate; RGC: retinal ganglion cell; ROS: reactive oxygen species; STAT3: signal transducer and activator of transcription 3; TGF: transforming growth factor; TNF: tumor necrosis factor.

The regulation of transitions between M1 and M2 microglial phenotypes is considered essential for reducing neuroinflammation and mitigating neuronal damage (Tang and Le, 2016; Zhang et al., 2020). **Table 3** presents a summary of major therapeutic strategies aimed at targeting microglial phenotypes. Current treatments for neuroinflammatory diseases such as multiple sclerosis provide examples of methods to modulate these phenotypes (Giunti et al., 2014). For instance, interferon-beta reduces neurotoxicity by regulating the JAK-STAT signaling pathway, thus inhibiting microglial release of pro-inflammatory cytokines such as TNF-α and IL-1β (Dasgupta et al., 2002; Kawanokuchi et al., 2004). Similarly, dimethyl fumarate diminishes neuroinflammation through activation of the Nrf2 antioxidant pathway, which suppresses M1 microglial activity and promotes a shift to the anti-inflammatory M2 phenotype (Schilling et al., 2006; Ghoreschi et al., 2011). Additionally, glatiramer acetate and fingolimod (FTY720) lower inflammatory cytokine release by adjusting microglial immune responses (Kim et al., 2004; Foster et al., 2007). Mesenchymal stem cell therapy also facilitates M2 polarization by secreting anti-inflammatory factors, thereby enhancing neuroprotection (Laroni et al., 2013). Together, these approaches highlight the therapeutic potential of modulating microglial phenotypes to alleviate neuroinflammation and offer potential benefits in glaucoma treatment.

**Table 3:** Therapeutic potential of modulating microglial phenotypes in glaucoma

Interventions	Mechanisms	Primary functions	References
Interferon-β	Modulates JAK-STAT pathway to inhibit pro-inflammatory cytokines (e.g., TNF-α and IL-1β)	Reduces neurotoxicity and inflammation	Dasgupta et al., 2002; Kawanokuchi et al., 2004
Dimethyl fumarate	Activates Nrf2 antioxidant pathway, suppresses M1 microglial activity	Promotes M2 polarization, alleviates neuroinflammation	Schilling et al., 2006; Ghoreschi et al., 2011
Glatiramer acetate	Modifies immune activity of microglia, reduces inflammatory cytokine release	Lowers neurotoxicity	Kim et al., 2004; Foster et al., 2007
Fingolimod (FTY720)	Regulates microglial activity states	Reduces cytokine release, mitigates inflammation	Kim et al., 2004; Foster et al., 2007
Mesenchymal stem cells	Secretes anti-inflammatory factors, promotes M2 polarization	Enhances neurotrophic support and neuroprotection	Laroni et al., 2013
TLR4/NF-κB pathway inhibition	Activation via DAMPs and PAMPs promotes M1 polarization	Reduces pro-inflammatory cytokines, mitigates neuroinflammation	Saijo and Glass, 2011; Eldahshan et al., 2019; Kumar, 2019
NLRP3 inflammasome inhibition	Modulates IL-1β and IL-18 secretion, promotes M1-to-M2 transition	Reduces neuroinflammation, protects neurons	Sui et al., 2020; Zhao et al., 2020
JAK/STAT pathway inhibition	Inhibits JAK2/STAT3 activation	Reduces pro-inflammatory response, alleviates neuroinflammation	Jones et al., 2015; Iwahara et al., 2017
AMPK and MAPK pathway activation	Improves metabolic status, promotes M2 polarization	Reduces inflammation, promotes tissue repair	Wang et al., 2018; Zhou et al., 2019; Plastira et al., 2020
miRNA regulation	Modulates transcription factors (e.g., CEBPα, PU.1) to influence microglial phenotype	Regulates inflammatory response, protects retinal ganglion cells	Fazi et al., 2005; Arora et al., 2013; Guedes et al., 2013; Butovsky et al., 2015

AMPK: 5& AMP-activated protein kinase; CEBP: CCAAT/enhancer‐binding protein; DAMPs: damage-associated molecular patterns; IL: interleukin; JAK: Janus kinase; MAPK: mitogen-activated protein kinase; NF-κB: nuclear factor ‘kappa-light-chain-enhancer’ of activated B-cells; NLRP3: NLR family pyrin domain containing 3; Nrf2: nuclear factor erythroid 2-related factor 2; PAMPs: pathogen-associated molecular patterns; STAT: signal transducer and activator of transcription; TLR: Toll-like receptor; TNF: tumor necrosis factor.

The phenotypic shift of microglia is governed by various signaling pathways (Li et al., 2021), including the TLR4/NF-κB, NLRP3 inflammasome, and JAK/STAT pathways. Activation of the TLR4/NF-κB pathway by DAMPs and pathogen-associated molecular patterns promotes M1 polarization, intensifying inflammation (Saijo and Glass, 2011; Eldahshan et al., 2019; Kumar, 2019). The NLRP3 inflammasome, once triggered by pathogen-associated molecular pattern and DAMP signals, leads to the release of IL-1β and IL-18; inhibiting this pathway encourages the transition from an M1 to an M2 phenotype, thereby reducing neuroinflammation (Sui et al., 2020; Zhao et al., 2020). Similarly, the JAK/STAT pathway modulates microglial polarization by controlling cytokine activity, with inhibition of JAK2/STAT3 shown to be a potential approach to managing neuroinflammation (Jones et al., 2015; Iwahara et al., 2017). Additionally, activation of the 5’ AMP-activated protein kinase and mitogen-activated protein kinase pathways positively influences microglial metabolism and supports M2 polarization (Wang et al., 2018; Zhou et al., 2019; Plastira et al., 2020). Targeting these key signaling pathways offers promising therapeutic potential for neuroprotection and anti-inflammatory treatments in glaucoma.

Endogenous molecular stressors can alter the activation phenotype of microglia (Zhang et al., 2018), with microRNAs (miRNAs) playing a critical role in regulating microglial development and function by modulating transcription factors such as CEBPα and PU.1 (Fazi et al., 2005; Arora et al., 2013; Guedes et al., 2013; Butovsky et al., 2015). Research indicates that resting microglia typically have low miR-155 and high miR-124 expression levels, while M1-polarized microglia show increased miR-155 and decreased miR-124 expression. In contrast, M2 microglia exhibit elevated miR-124 and reduced miR-155 expression (Ponomarev et al., 2013; Porta et al., 2015). Furthermore, miRNAs are capable of modulating microglial inflammatory responses, offering potential pathways for neuroprotection and tissue repair in glaucoma. Therapeutic approaches using miRNA-based strategies may provide targeted regulation of microglial phenotypes, thus protecting RGCs and enhancing treatment options for glaucoma.

Microglial phenotypic transformation has gained attention as a promising therapeutic target in neurodegenerative diseases, including glaucoma. Targeting these mechanisms opens new pathways for reducing neuroinflammation and safeguarding RGCs. Investigating the application of these drugs and related strategies in glaucoma could enhance the therapeutic potential of microglial phenotype modulation, offering broader possibilities for treating neurodegenerative conditions.

## Conclusion

Neuroinflammation, neurodegeneration, and neuroregeneration are central themes in retinal pathology research, with neuroinflammation playing a particularly intricate role in glaucoma. Microglial activation is pivotal in RGC dysfunction and degeneration, where it can both aggravate neuronal damage and, in certain contexts, facilitate tissue repair and regeneration. As such, regulating microglial activity presents a compelling therapeutic target for innovative treatments in CNS injuries. Gaining insight into the dual role of microglia in glaucoma is essential for unraveling the disease’s complex pathophysiological mechanisms and for the development of novel therapeutic approaches, potentially enhancing prognosis and outcomes for glaucoma patients.

## Data Availability

*Not applicable*.
